# Advancing Exposure Index in Radiology for Optimized Imaging, Accuracy, and Future Innovations

**DOI:** 10.7759/cureus.80819

**Published:** 2025-03-19

**Authors:** Petros I Soulis, Periklis Papavasileiou, Athanasios Bakas, Eleftherios Lavdas, Nikolaos Stogiannos

**Affiliations:** 1 Department of Biomedical Sciences, University of West Attica, Athens, GRC; 2 Department of Radiology, General Hospital of West Attica, Athens, GRC; 3 Division of Midwifery and Radiography, University of London, London, GBR

**Keywords:** artificial intelligence, digital radiography, exposure index, quality control, radiation dose optimization, standardization

## Abstract

Exposure index (EI) is a critical parameter in digital radiography, providing a quantitative measure of the radiation dose received by the detector. This review examines the significance of EI, methods for its determination, influencing factors, and clinical implications. Additionally, it explores challenges in standardization efforts and the role of emerging technologies, particularly artificial intelligence (AI), in optimizing exposure management.

A comprehensive review of literature published over the last two decades was conducted using databases such as PubMed, ScienceDirect, and Google Scholar. Studies addressing EI measurement, clinical applications, and advancements in exposure monitoring technology were analyzed. Guidelines from the International Electrotechnical Commission (IEC), the American Association of Physicists in Medicine (AAPM), and the European Federation of Organizations for Medical Physics (EFOMP) were also reviewed to assess standardization efforts and best practices.

Findings highlight the importance of EI in radiation dose optimization and quality control. Despite standardization initiatives, variations persist across manufacturers and imaging systems due to factors such as patient characteristics, beam energy, detector sensitivity, and post-processing algorithms. Artificial intelligence-driven exposure monitoring systems have shown promise in enhancing EI accuracy and enabling real-time dose adjustments.

Artificial intelligence technologies have the potential to revolutionize EI utilization by enabling automated exposure optimization, real-time monitoring, and predictive analytics. Future efforts should focus on refining AI algorithms, ensuring cross-platform standardization, and enhancing radiographer training to fully integrate AI into EI-based radiation safety protocols.

## Introduction and background

The transition from conventional film-screen radiography to digital radiography has necessitated the development of standardized metrics to assess radiation exposure. In film-based imaging, optical density serves as a subjective indicator of exposure; however, digital imaging requires objective and reproducible measures to ensure accurate radiation dose management. The exposure index (EI) has emerged as a critical parameter in digital radiography, providing a numerical representation of the radiation dose received by the image receptor during an imaging procedure [1,2].

Accurate utilization of EI is essential for maintaining diagnostic image quality while adhering to the ALARA (short for as low as reasonably achievable) principle, a fundamental guideline in radiology aimed at minimizing patient radiation exposure without compromising diagnostic efficacy. A well-calibrated EI assists radiographers and radiologists in evaluating whether exposure settings are appropriate, thereby enhancing patient safety and reducing unnecessary radiation doses [3,4].

Despite the recognized importance of EI, its clinical implementation faces several challenges. Variability in EI calculation methodologies across different digital radiography systems, inconsistencies in manufacturer-specific EI reporting, and a lack of universal standardization have hindered widespread adoption. Moreover, the misinterpretation of EI values by radiographers can lead to either excessive radiation exposure or suboptimal image quality, underscoring the need for improved training and standardization [5].

Recent advancements in artificial intelligence (AI) and automated exposure monitoring present promising solutions to these challenges. Artificial intelligence-driven exposure optimization tools have the potential to enhance EI accuracy, provide real-time feedback, and facilitate standardized exposure management across different imaging systems. By integrating AI technologies, radiology departments can improve consistency in EI utilization and ensure better adherence to radiation safety protocols.

This review aims to provide a comprehensive analysis of EI in digital radiography by examining its significance, measurement techniques, clinical implications, and ongoing standardization efforts. Additionally, the review explores challenges associated with EI implementation and the role of emerging technologies in optimizing exposure management. Through a critical evaluation of existing literature, this study seeks to offer insights into the future of EI standardization and its integration into clinical practice.

## Review

Definition and importance of EI

The EI is a standardized numerical value that represents the amount of radiation exposure received by a digital radiographic detector [6]. Unlike conventional film-based radiography, where exposure is inferred from optical density, digital systems require objective parameters to monitor and optimize radiation dose levels [7]. The EI plays a vital role in radiographic quality assurance by providing real-time feedback on exposure settings, allowing radiographers to make necessary adjustments to maintain image quality while minimizing patient radiation dose [8]. 

Standardization of EI 

Efforts to standardize EI have been led by the International Electrotechnical Commission (IEC) and the American Association of Physicists in Medicine (AAPM), with the IEC 62494-1 standard establishing guidelines for EI measurement and interpretation [7,9]. However, variations still exist among different imaging manufacturers, leading to inconsistencies in EI reporting. Some digital radiography systems display EI values differently, making it challenging for radiographers to interpret and compare exposure levels across different equipment. Standardizing EI across all imaging systems remains an ongoing challenge, with collaborative efforts needed from regulatory bodies, professional organizations, and imaging manufacturers [10].

Factors influencing EI

The EI is a crucial parameter in diagnostic radiology, providing an indirect measure of the radiation dose a patient receives during an imaging procedure [3]. While not a direct measure of patient dose, it serves as a surrogate indicator of exposure, reflecting the balance between the radiation delivered and the image quality obtained [11]. The concept of EI has gained importance in the field of radiology, especially with the advent of digital radiography, where it plays a key role in ensuring that patients receive the lowest possible radiation dose while still obtaining diagnostic-quality images [12]. However, several factors influence the EI value, including patient-specific variables such as body mass index (BMI), age, gender, and anatomical size, as well as technical factors related to radiographic techniques like kilovolt peak (kVp), milliampere-seconds (mAs), and source-to-image distance (SID). These variables contribute to the complexity of EI interpretation, as they all affect how much radiation is required to produce an image with sufficient quality and detail [13].

Body mass index and EI

Recent research highlights the importance of patient size in optimizing radiation dose in digital radiography. A study in radiography found that overweight and obese patients receive significantly higher radiation doses than those with normal BMI, emphasizing the need for BMI-based protocol adjustments [14]. Another study in the Journal of Applied Clinical Medical Physics proposed technique charts using patient weight and height to ensure consistent exposure levels in portable chest and abdominal radiography, benefiting both adults and pediatric patients [15]. Additionally, a phantom study demonstrated that entrance surface dose (ESD) increases with body thickness while maintaining a constant EI, suggesting that EI alone may not fully reflect dose variations [16].

Further research reinforces the significance of BMI in exposure optimization. A study by Soulis et al. (2024) investigated the correlation between EI and BMI in patients undergoing routine posterior-anterior (PA) chest radiographs. The research encompassed 805 patients and analyzed various factors, including EI, BMI, ESD, source-to-object distance (SOD), age, and gender. The findings revealed that an increase in BMI was associated with a decrease in EI. Additionally, higher ESD correlated with increased EI, except in patients with very low or very high BMI, where no significant relationship was observed. These results suggest that BMI influences EI, and adjustments in radiographic parameters may be necessary to maintain optimal image quality across different patient body compositions [13].

Further research by Dolenc et al. (2022) examined the impact of BMI on patient radiation dose in general radiography. The study concluded that higher BMI leads to increased radiation doses due to the necessity for higher exposure parameters to achieve diagnostic image quality. This underscores the importance of tailoring exposure settings based on patient BMI to ensure both image quality and patient safety [17].

Moreover, Darcy et al. (2015) explored variations in exposure factor selection by radiologic technologists for patients with varying BMI. The study found significant differences in exposure parameter choices, highlighting the need for standardized guidelines and training to assist technologists in making informed decisions that account for patient BMI, thereby optimizing EI and minimizing unnecessary radiation exposure [18].

Body mass index is a measure of body fat based on an individual’s weight and height, and it plays a crucial role in determining how much radiation is needed to obtain an image [17]. Patients with higher BMI, particularly those with a high amount of adipose tissue, generally require a higher dose of radiation [19]. This is because increased body mass requires more radiation to penetrate the tissue and produce an image of diagnostic quality [20]. For example, an obese patient would typically need higher mAs and kVp settings to ensure that the X-rays can adequately penetrate the body and produce a clear image, especially when imaging dense areas such as the abdomen or pelvis. In contrast, patients with lower BMI require less radiation, as there is less tissue for the X-rays to penetrate. This variation in exposure needs based on BMI directly impacts the EI value, as higher radiation doses lead to higher EI values. Without proper adjustments to exposure settings for patients with high BMI, there is a risk of overexposure, which could increase unnecessary radiation dose, or underexposure, which could result in inadequate image quality and diagnostic challenges [21].

Body mass index significantly influences EI, requiring tailored exposure settings to balance image quality and radiation safety. Studies show higher BMI decreases EI while increasing radiation dose needs, yet extreme BMI cases show inconsistent EI correlations. Variability in technologists' exposure choices highlights the need for standardized guidelines. While BMI-based adjustments improve diagnostics, over-reliance on set protocols may overlook individual patient differences. The challenge lies in optimizing exposure without excessive radiation, especially for obese patients. Future research should refine dynamic exposure strategies rather than rigid parameter adjustments.

Kilovolt peak, milliampere-seconds, and EI

Another significant factor that influences EI is the kVp, which determines the energy of the X-ray beam. The kVp determines the energy and penetrative ability of the X-ray photons produced [22]. Higher kVp settings result in X-rays with greater energy, enhancing their ability to penetrate denser tissues. This increased penetration can lead to a higher EI, as more photons reach the detector [23]. Conversely, lower kVp settings produce X-rays with less energy, resulting in reduced penetration and a lower EI due to fewer photons reaching the detector [24].

A study published in the Journal of Medical Imaging and Radiation Sciences explored the reliability of the EI across a range of kVp and mAs values [25]. The findings indicated that while EI is a useful tool for assessing exposure, its reliability can be influenced by variations in kVp and mAs settings. This underscores the importance of understanding the relationship between EI and kVp to ensure accurate dose assessment and optimal image quality.

Other studies evaluated the correlation between radiographic exposure techniques and the entrance surface air kerma (ESAK) using EI from computed and digital radiography. The results demonstrated a strong correlation between EI and ESAK, suggesting that EI can be a reliable indicator of patient dose. However, the study also found a poor correlation between EI and kVp, indicating that while EI is influenced by exposure factors, it may not directly reflect changes in kVp [26].

Similarly, the mAs is another critical technical factor influencing EI. Studies have demonstrated a direct linear relationship between mAs and EI. Increasing the mAs results in a proportional increase in the radiation dose to the detector, leading to a higher EI [27]. Conversely, decreasing the mAs reduces both the radiation dose and the EI. This linearity implies that careful adjustment of mAs can control the EI, thereby influencing image quality and patient exposure [28-30].

For instance, a study investigating the reliability of EI across various kVp and mAs settings found that EI values increased consistently with higher mAs, confirming the direct correlation between mAs and EI. The study emphasized the importance of understanding this relationship to ensure appropriate exposure levels and maintain image quality [25].

Both kVp and mAs significantly influence EI, yet their effects differ, with kVp’s impact on EI being inconsistent. While EI is a useful exposure indicator, its reliability fluctuates with kVp and mAs variations, questioning its precision in dose assessment. The strong EI-ESAK correlation supports EI as a dose metric, but its weak correlation with kVp suggests limitations in reflecting beam energy changes. The linear mAs-EI relationship simplifies exposure control but risks overlooking nuanced patient needs. Over-reliance on EI may lead to suboptimal imaging decisions if not balanced with other quality factors. Future studies should refine EI’s role beyond basic exposure correlations for personalized dose optimization.

Source-to-image distance and EI

Source-to-image distance also plays an important role in determining the radiation exposure and consequently the EI [31]. Source-to-image distance refers to the distance between the X-ray tube and the image receptor. According to the inverse square law, the intensity of radiation decreases as the distance from the source increases. Therefore, a greater SID results in lower radiation intensity reaching the patient and the image receptor, while a shorter SID increases the intensity. To compensate for the lower radiation intensity at longer distances, radiographers may need to adjust the exposure settings (such as increasing mAs or kVp) to maintain adequate image quality. Variations in SID can thus influence the EI by affecting the amount of radiation delivered to the patient [13,32]. Proper calibration of SID is essential to ensure that the radiation dose is optimized for each imaging procedure, as inconsistent SID measurements can lead to inaccurate EI values and potential over- or under-exposure of the patient. Since EI is influenced by the actual radiation reaching the detector, also SOD variations indirectly affect EI by altering beam intensity and attenuation. A shorter SOD increases radiation intensity at the detector, potentially increasing EI, while a longer SOD decreases EI unless compensated by increased exposure settings [13].

Source-to-image distance and SOD affect radiation exposure and EI due to the inverse square law, requiring exposure adjustments for image consistency. However, reliance on SID calibration can introduce variability, potentially leading to inconsistent EI values and patient exposure risks.

Age, gender, and EI

Age and gender are additional factors that influence the EI, though their effects are somewhat more indirect. Age-related changes in tissue composition, such as the decrease in bone density with age or the differences in fat and muscle mass between genders, can alter the way radiation is absorbed by the body [33,34]. For example, pediatric patients generally require lower radiation doses because of their smaller size and reduced tissue density compared to adults. In contrast, older patients may require slightly higher doses due to the loss of bone density, which affects the way radiation interacts with the body. Gender differences, particularly in body composition, also impact radiation absorption. Women typically have a higher percentage of body fat than men, which can lead to differences in radiation dose requirements [13,35]. These demographic variations highlight the need for dose optimization based on patient characteristics, ensuring that the EI values are adjusted to account for age and gender differences.

A study evaluating EI values in PA chest X-rays found a weak negative correlation between patient age and EI (r = -0.084, p = 0.015). This suggests that as patient age increases, EI values tend to decrease slightly. These findings indicate that younger patients (15-29 years) had higher mean EI values compared to older age groups. The decrease in EI with age could be attributed to factors such as changes in tissue composition and density, which affect X-ray attenuation [34].

The same study observed EI values in male and female patients undergoing PA chest X-rays. The mean EI for females was 172.86 ± 92.50, while for males, it was 173.48 ± 82.25. The difference in EI between genders was not statistically significant (p=0.237), suggesting that gender does not have a substantial impact on EI values in this context [34].

In contrast, another study reported that female patients recorded significantly higher median EI values than male patients for various examinations, including chest PA and lateral views. This discrepancy highlights the need for further research to understand the underlying causes, which may include differences in body composition, breast tissue density, or variations in radiographic technique [13].

Despite its usefulness, there are several challenges in the consistent application of EI. One of the primary issues is the lack of standardization in the way EI is reported and interpreted across different imaging systems. Different manufacturers use different algorithms to calculate and report EI values, which can lead to inconsistencies in the data [31]. This variability in EI reporting can create confusion for radiographers and radiologists, making it difficult to assess whether a particular EI value indicates optimal radiation exposure for a given patient. Furthermore, many radiographers may not be fully trained to interpret EI values accurately or understand their significance in terms of patient safety. This lack of training and standardization can hinder the widespread adoption of EI as a reliable tool for dose optimization [35].

Age and gender subtly influence EI through tissue composition changes, but their impact remains inconsistent. While older patients may require higher doses, EI’s weak age correlation questions its reliability as an exposure guide. Gender differences in EI remain debated, with conflicting studies highlighting potential biases in measurement or technique. The lack of EI standardization across imaging systems leads to inconsistencies, limiting its clinical reliability. Insufficient radiographer training further complicates EI interpretation, reducing its effectiveness in dose optimization. Future efforts should focus on standardizing EI metrics and enhancing technologist education for consistent, patient-specific radiation management.

Filtration and EI

Filtration involves placing materials, typically metals like aluminum or copper, in the path of the X-ray beam to remove low-energy photons that contribute to patient dose without enhancing image quality. The interplay between EI and filtration is crucial for achieving optimal imaging outcomes while ensuring patient safety.

Adding filtration to the X-ray beam modifies its energy spectrum by absorbing low-energy photons, resulting in a "harder" beam with higher average energy. This process reduces the entrance skin dose to the patient and can influence the EI. It's important to note that the EI is calibrated based on specific beam qualities, including kVp and filtration. Changes in filtration alter the beam's half-value layer (HVL), which can affect the EI's accuracy if not properly accounted for during system calibration. The AAPM Task Group 116 emphasizes the need for standardizing the EI to reflect consistent detector exposures across varying beam qualities [7].

Filtration enhances beam quality by removing low-energy photons, reducing patient dose while influencing EI. However, EI calibration depends on specific beam parameters, and filtration changes can affect its accuracy. Variability in HVL due to filtration adjustments raises concerns about EI consistency across systems. AAPM calls for EI standardization, yet implementation remains inconsistent in practice. Overlooking filtration’s impact on EI calibration may lead to misinterpreted exposure levels. Future research should refine EI frameworks to account for dynamic filtration effects, ensuring reliable dose assessment.

Entrance skin dose, dose area product, and EI

While EI is not a direct measure of patient dose, studies have explored its correlation with patient dosimetric parameters such as ESD and dose-area product (DAP).

A study by Erenstein et al. investigated the relationship between EI and DAP using anthropomorphic phantoms for pelvis and chest examinations. The researchers varied exposure parameters, including kVp and mAs, and analyzed the resulting EI and DAP values. They found a strong linear correlation between EI and DAP, with R-squared values exceeding 0.987 across different exposure settings. This suggests that as the exposure to the detector increases, both EI and DAP increase proportionally. However, the study also noted that factors such as region of interest (ROI) placement and body habitus can influence this relationship, indicating the need for careful consideration in clinical practice [36].

Soulis et al. conducted a study to assess the correlation between EI and ESD in chest PA radiographic projections. The findings revealed that an increase in ESD was generally associated with an increase in EI. However, this relationship was not statistically significant in patients with lower or higher BMI, suggesting that body composition can affect the correlation between EI and ESD. The study concluded that while EI can serve as a useful indicator, it should be interpreted alongside other parameters to ensure accurate dose assessment [13].

Yoon et al. conducted a phantom study to assess the relationship between EI and ESD in digital chest radiography. The study revealed that while ESD increased with phantom thickness, the EI remained relatively constant due to the automatic exposure control (AEC) system maintaining consistent image receptor exposure. This indicates that EI may not reliably reflect changes in patient dose under certain conditions, emphasizing the importance of considering patient body habitus when using EI for dose optimization [37,38].

The EI correlates with dosimetric parameters like DAP and ESD, but its reliability varies with patient body habitus and exposure settings. While studies show a strong EI-DAP relationship, factors like ROI placement complicate its clinical utility. The EI-ESD correlation weakens at extreme BMI ranges, questioning EI’s consistency across diverse patients. The AEC systems can maintain EI stability despite increasing ESD, highlighting EI’s limitations in direct dose assessment. Over-reliance on EI without considering patient-specific factors risks misinterpreted exposure levels. Future research should refine EI interpretation for more accurate, individualized dose optimization.

Type of examination, anatomical area, and EI

In addition to patient-related factors, the type of imaging examination and the anatomical area being imaged also influence the EI. Different body parts require different exposure settings depending on their density and thickness [16]. For example, imaging of the chest or abdomen typically requires different exposure settings, with the chest requiring a higher kVp and lower mAs compared to the abdomen. Similarly, imaging smaller body parts, such as the hands or feet, requires less radiation than larger parts like the pelvis or spine. The imaging technique used, whether it involves standard radiography, fluoroscopy, or mammography, also affects the radiation dose required and, consequently, the EI. Each technique has its own set of exposure parameters, and the EI values will vary depending on how these parameters are adjusted to meet the specific clinical needs of the examination [39].

Different imaging examinations require varying exposure parameters due to the distinct characteristics of the tissues and structures being assessed. For instance, a chest radiograph necessitates different exposure settings compared to an abdominal radiograph, primarily because of differences in tissue density and composition [40]. These variations directly impact the EI, as the amount of radiation needed to achieve a diagnostically useful image differs between examination types [41]. A study by Seibert and Morin (2011) emphasizes that the EI should be tailored to specific examinations to ensure both image quality and patient safety [3].

The anatomical area under examination significantly affects the EI due to variations in tissue density, thickness, and composition. For example, imaging of denser structures like the pelvis requires higher radiation doses compared to less dense areas like the chest. This is because denser tissues attenuate more X-rays, necessitating increased exposure to obtain a clear image. The AAPM highlights that understanding these anatomical differences is vital for setting appropriate exposure parameters and interpreting EI values accurately [7].

A significant challenge in utilizing EI effectively is the lack of standardization across different imaging systems and insufficient training among radiographers. Variations in EI calibration and reporting methods can lead to confusion and misinterpretation, potentially compromising patient safety. A study published in the Journal of Medical Imaging and Radiation Sciences found that many radiographers may not be fully trained to interpret EI values accurately or understand their significance in terms of patient safety. This lack of training and standardization can hinder the widespread adoption of EI as a reliable tool for dose optimization [42].

Effective application of the EI hinges on a deep understanding of how imaging examinations and anatomical variations influence exposure parameters. To maximize its potential, future efforts should focus on AI-driven training tools and adaptive protocols that personalize EI interpretation based on specific imaging contexts. Standardized, data-driven education programs can enhance radiographers' competency, ensuring consistent EI usage for improved patient safety and optimized image quality.

A critical challenge in EI application is the potential for misinterpretation when comparing values across different examinations and anatomical regions. While EI provides a quantitative measure of exposure, it does not inherently account for the varying diagnostic requirements of different tissues. A standardized approach that incorporates anatomical weighting factors or AI-driven adjustments could refine EI interpretation, making it more clinically relevant. Additionally, reliance on EI alone without considering other dose metrics, such as DAP or ESD, may lead to oversimplified assessments of radiation exposure. Future advancements should focus on integrating EI with multi-parameter dose evaluation models to enhance its precision and applicability in diverse clinical scenarios.

Modulation transfer function (MTF)​​​​​, detective quantum efficiency (DQE)​​​​​, and EI

The MTF and DQE are critical parameters in digital radiography that directly impact image quality and the EI. The MTF measures a system’s ability to accurately transfer contrast from the object being imaged to the final image, essentially defining the system’s spatial resolution. A higher MTF ensures that fine details are well-preserved, reducing the need for increased radiation exposure to maintain image clarity. On the other hand, DQE quantifies how efficiently an imaging system converts incoming X-ray photons into a useful image while minimizing noise. A detector with a high DQE requires less radiation to produce a high-quality image, leading to a lower EI, whereas a system with low DQE may need higher exposure settings, increasing EI. Both MTF and DQE play crucial roles in optimizing radiation dose and image quality, reinforcing the importance of well-calibrated imaging systems to ensure diagnostic accuracy while keeping patient exposure as low as possible [3].

Heel effect and EI

The anode heel effect is a phenomenon in radiography where the intensity of X-rays emitted from the anode varies along the anode-cathode axis, resulting in a decrease in radiation intensity toward the anode side. This occurs because X-rays produced within the anode must traverse varying amounts of target material before exiting; photons emitted toward the anode side pass through more material, leading to greater absorption and reduced intensity compared to those emitted toward the cathode [43].

This variation in X-ray intensity can lead to non-uniform image quality, particularly in digital radiography. A study by Chou (2021) evaluated this non-uniformity using a novel circular step-wedge phantom and normalized mutual information metrics. The findings indicated significant changes in image quality metrics with different orientations, highlighting the sensitivity of digital radiographs to the anode heel effect [44].

The anode heel effect can also influence the contrast-to-noise ratio (CNR) in computed radiography. The CNR is a key metric in medical imaging that quantifies the visibility of a structure relative to its background by measuring the difference in contrast between an object and surrounding noise levels. A higher CNR indicates a clearer distinction between anatomical structures, improving diagnostic accuracy, while a lower CNR means that an image may appear grainy or have poor visibility of fine details. The anode heel effect influences CNR in computed radiography (CR) by causing variations in X-ray intensity across the image field, leading to non-uniform contrast and potential reductions in image quality. In areas where X-ray intensity is lower (toward the anode side), image contrast may decrease, impacting the CNR and potentially affecting diagnostic interpretation. Research by Ratini et al. (2023) examined the application of the anode heel effect with a step-wedge phantom across various X-ray tube voltages. The study found that higher tube voltages resulted in lower CNR values, suggesting that the anode heel effect can affect image contrast and quality [45].

In clinical practice, the anode heel effect can be utilized to optimize image quality by positioning the patient such that thicker body parts are aligned with the cathode side, where X-ray intensity is higher. For instance, in thoracic spine radiography, aligning the anode toward the cranial part of the patient has been shown to improve image quality for specific vertebrae [46].

In summary, the anode heel effect significantly impacts image quality in radiography by causing variations in X-ray intensity across the image field. Understanding and managing this effect is crucial for accurate image interpretation and patient safety.

Artificial intelligence and EI

The integration of AI into radiology has the potential to enhance the interpretation of EI values, thereby improving patient safety and dose optimization. Artificial intelligence algorithms, particularly deep learning models, excel at recognizing complex patterns in imaging data and can provide quantitative assessments that assist radiographers in making informed decisions [47].

A study highlighted that AI can optimize personnel allocation and scanner usage, leading to reduced radiation exposure and improved efficiency in radiological practices [48]. By analyzing large datasets of radiographic images, AI systems can learn to identify optimal exposure parameters, ensuring that images are of diagnostic quality while minimizing patient dose.

Moreover, AI can assist in standardizing EI values across different imaging systems and protocols. The lack of standardization in EI has been a challenge in digital radiography, leading to inconsistencies in image quality and patient dose. Artificial intelligence algorithms can be trained to recognize and adjust for these discrepancies, promoting uniformity in radiographic practices [49].

Beam restriction, achieved through collimation, plays a significant role in influencing EI values and, consequently, patient safety. Beam restriction limits the size of the X-ray field to the area of interest, reducing the volume of irradiated tissue and minimizing scatter radiation. This reduction in scatter enhances image contrast and can affect the EI. A more collimated (restricted) beam results in less scatter reaching the detector, potentially leading to a lower EI, which might prompt radiographers to increase exposure parameters unnecessarily if not properly understood.

A study by Yasumatsu et al. examined the effect of X-ray beam quality on the determination of EI and found that variations in beam quality significantly impact EI values. The study indicated that the effect of variations in X-ray beam qualities in the recurrence quantification analysis (RQA) series on the EI was significantly greater than the effect of grids, highlighting the importance of consistent beam quality and proper collimation in maintaining accurate EI readings [50].

Furthermore, the AAPM Task Group 116 emphasizes that inadequate or excessive exposure is manifested as higher or lower image noise levels instead of as light or dark images. In digital imaging, brightness and contrast are often determined entirely by digital postprocessing of the acquired image data. Overexposure and underexposure are not readily recognizable. As a result, the patient dose has a tendency to gradually increase over time after a department converts from screen/film-based imaging to digital radiographic imaging [7]. The application of AI in interpreting and standardizing EI values holds promise for enhancing patient safety through improved dose optimization and consistency in radiographic imaging.

While AI holds promise for optimizing exposure protocols, its implementation requires careful consideration of factors such as data quality, algorithm validation, and integration into existing workflows. AI systems can automate exposure parameter adjustments by analyzing patient-specific factors such as BMI to recommend optimal settings that achieve appropriate EI values. Real-time feedback mechanisms can further enhance image acquisition by allowing radiographers to modify exposure parameters on the fly, ensuring that EI remains within the desired range. Additionally, AI-driven quality assurance and monitoring can continuously track EI values across various examinations, identifying trends and deviations to facilitate proactive adjustments to imaging protocols [49]. Ongoing research and collaboration between radiographers, radiologists, and AI developers are essential to fully harness AI's potential in this context. Although direct studies on AI’s role in EI optimization are still emerging, advancements in AI applications within radiology indicate a promising avenue for future research and development.

Future directions and potential of EI

The future of the EI in digital radiography lies in overcoming its current limitations through technological innovation, standardization, and enhanced clinical integration. While EI serves as a valuable metric for assessing radiation exposure, its variability across different imaging systems and patient factors highlights the need for improved consistency and usability. 

Emerging advancements in AI hold significant potential for revolutionizing EI applications. Artificial intelligence-driven exposure monitoring can facilitate real-time adjustments, automate exposure parameter recommendations, and standardize EI interpretation across diverse imaging platforms. Predictive analytics could further refine radiation dose optimization by analyzing historical data and patient profiles, enabling personalized imaging protocols [13]. 

A key component of this advancement is the integration of EI values into Picture Archiving and Communication Systems (PACS) using Digital Imaging and Communications in Medicine (DICOM). The EI values are embedded within DICOM metadata under specific tags, allowing radiologists and technologists to track radiation exposure trends over time. This integration enhances quality assurance, dose optimization, and standardization of imaging protocols across different radiology departments. By storing EI values alongside patient images, healthcare providers can analyze exposure trends, identify inconsistencies, and make data-driven adjustments to imaging techniques, ensuring optimal image quality with minimal radiation dose.

Standardization remains a critical challenge, requiring continued collaboration among regulatory bodies, manufacturers, and healthcare institutions. Establishing universal guidelines for EI interpretation, reporting, and cross-platform compatibility will ensure consistent application and enhance patient safety. Additionally, expanding training programs for radiographers and radiologists through AI-assisted tools, hands-on workshops, and simulation-based learning will promote a more comprehensive understanding of EI. 

Future research should focus on validating the correlation between EI and actual radiation dose across various populations and modalities. Additionally, integrating EI with other dose-related parameters, such as DAP and ESD, will provide a more holistic approach to radiation dose assessment. The goal is to transition EI from a retrospective evaluation tool to a real-time decision-making asset, ensuring optimal image quality while minimizing radiation exposure. Ultimately, the potential of EI lies in its evolution into a dynamic, AI-enhanced, and standardized metric that supports personalized, safe, and efficient radiographic imaging practices [51].

## Conclusions

The EI plays a pivotal role in digital radiography by providing a quantifiable measure of radiation exposure, facilitating dose optimization, and ensuring adherence to radiation safety protocols. Despite efforts from organizations such as the IEC and the AAPM, challenges persist in standardizing EI across different manufacturers and imaging systems. Variability in EI reporting, differences in detector sensitivity, and inconsistent interpretation among radiographers highlight the need for enhanced training, standardized guidelines, and improved calibration methods. Additionally, patient-specific factors such as body mass index, anatomical region, and technical parameters like beam energy and detector sensitivity further complicate EI utilization, necessitating individualized exposure adjustments.

The integration of AI and automated exposure monitoring systems presents a promising solution to address these limitations. AI-driven technologies can enhance the accuracy and consistency of EI interpretation, provide real-time feedback for exposure adjustments, and facilitate cross-platform standardization. Future research should focus on refining AI algorithms, improving interoperability between imaging systems, and developing comprehensive training programs to optimize EI-based dose management. By leveraging technological advancements and reinforcing standardized protocols, the radiology community can ensure that EI serves as a reliable tool for enhancing diagnostic accuracy, improving patient safety, and advancing radiation protection practices in digital radiography.
